# Supplementary material for: Early White Matter Microstructure Alterations in Infants with Down Syndrome
Source: medRxiv. 2025 Feb 27:2025.02.26.25322913. Preprint. [Version 2] doi: 10.1101/2025.02.26.25322913 (PMC11888504; doi:10.1101/2025.02.26.25322913)
Supplement: Supplement 1 [file media-1.pdf]

Supplemental Material

Azrak O, Garic D, Nasir A, et al; for Infant Brain Imaging Study (IBIS) Network. Early White Matter Microstructure Alterations in Infants with Down Syndrome.

eMethods. Supplementary Methods

eFigure 1. An example of the susceptibility artifact is seen bilaterally in the temporal poles on high b-value (A) and low b-value (B) DWI.

eTable 1. Index of all tracts examined in secondary analyses.

eTable 2. Results of Multivariate analyses of variance comparing DTI and NODDI parameters in the tracts of interest between DS and control infants. (****p*-value is <0.0001, ***p*-value is 0.01 - 0.001, **p*-value is 0.05 - 0.01).

eFigure 2. Visualization of the significant *p*-values along the tracts generated using Slicer. (A) displays significant regions of RD in the bilateral CST, (B) highlights significant RD in the left SLF II and ILF, and (C) shows significant FA in the right IFOF and UNC. Abbreviations: IFOF, inferior fronto-occipital fasciculus; ILF, inferior longitudinal fasciculus; SLF, superior longitudinal fasciculus; CST, corticospinal tract; UNC, uncinate fasciculus. FA, fractional anisotropy; RD, radial diffusivity.

eTable 3. Full statistical results across all tracts**.**

eMethods

IBIS Infant Studies Background:

The study is part of the Infant Brain Imaging Study (IBIS), an ongoing multisite longitudinal study, collecting developmental behavioral and neuroimaging data on infants with neurodevelopmental conditions. The IBIS–DS studies infants with and without DS between the ages of 6 and 24 months. The IBIS–Early Prediction (IBIS-EP) studies infants at elevated familial likelihood for ASD between the ages of 6 and 24 months.

Data collection occurred at five sites: The Children's Hospital of Philadelphia (CHOP), The University of Minnesota (UMN), The University of North Carolina at Chapel Hill (UNC), The University of Washington (UW), and Washington University in St. Louis (WashU). Infants were recruited, scanned, and examined between March 2019 and May 2024.

Exclusion Criteria:

Exclusion criteria for all infants included: a diagnosis or physical signs of known genetic conditions (other than DS in DS infants), significant medical conditions the could impact growth, development, cognition, or sensory function (except for conditions commonly associated with DS in DS infants), birth weight <2500 grams, gestational age <34 weeks for DS infants or <36 weeks for control infants, history of significant perinatal complications, prenatal exposure to neurotoxins, maternal gestational diabetes requiring medication, contraindication for MRI, and families whose primary language is not English, due to requirements for cognitive assessments.

MRI Processing and Quality Control:

All native data underwent automated quality control (QC) using DTIPrep1.2.9[1] to remove any poor-quality DWI volumes. A preliminary visual QC using 3D Slicer 5.1.0 was then performed by a trained rater who visually assessed the quality of the remaining volumes, excluding any scan with significant motion artifact, consistent signal dropout, or surviving volumes below a predetermined threshold (80%). For participants with multiple series, the rater selected the highest-quality series.

Following automatic and preliminary QC, the remaining volumes were corrected for susceptibility, motion, and eddy current artifacts jointly via topup and eddy_openmp in FSL 6.0.3.[2] In the same procedure, the remaining artifact/outlier data were corrected/interpolated in FSL 6.0.3.[3] Brain masks were automatically generated as part of this procedure as these are necessary for the FSL processing. The automatic brain masks were computed via FSL bet applied to the (susceptibility corrected) average baseline/B0 image. All brain masks were reviewed and edited by trained raters.

A final visual QC was conducted by a trained rater who assessed the individual DWI volumes using DMRIPrep 0.5.0. DWI volumes with significant motion artifacts or signal dropouts were excluded. The rater also evaluated each scan's tractography based on the general expected anatomy, tract symmetry, and major tract integrity. Only scans with expected tractography and >80% surviving gradients passed.

DTI Processing:

A study-specific diffeomorphic, unbiased DTI atlas was created using the UNC-Utah National Alliance for Medical Image Computing DTI framework.[4] Brain-masked Diffusion Tensor Images (weighted least squares estimation of tensors) of 70 subjects were used to create the atlas. Then, each individual subject DTI were mapped to atlas space using nonlinear, diffeomorphic pair-wise registration, with visual inspection to verify registration accuracy.

Susceptibility Artifact Removal:

To remove the artifact from the analyses, the region containing the artifact was manually segmented in 10 individual DWI scans, with the mask then propagated to the DWI atlas for the entire sample. Any segments of tracts affected by the artifact were excluded from the analyses. Tracts affected were the tapetum of the corpus callosum, bilateral ILFs, bilateral fornices, and bilateral UNCs. There was no significant group difference in the occurrence rate of the artifact (X^2=1.44, p=0.23).

White Matter Metrics Extraction:

Fiber tractography was performed using AutoTract followed by manual refinement based on established fiber definitions.[5] The resulting deformation fields mapped atlas fibers to individual subject spaces, where diffusion tensor and NODDI metrics were extracted at equidistant points along each fiber tract. Diffusion tensors were estimated via a weighted least squares approach in Dmriprep[6], DTI properties—FA, AD, RD, MD—were extracted from the estimated tensors. NODDI properties—neurite density index (NDI) and orientation dispersion index (ODI)—were generated using Accelerated Microstructure Imaging via Convex Optimization (AMICO).[7]

For quality control, we excluded subjects whose tract-specific FA profiles correlated poorly ($r < 0.70$) with the population average, as this typically indicates suboptimal DTI-to-atlas alignment. Final tract metrics were computed by averaging the respective diffusion parameters along each fiber tract.

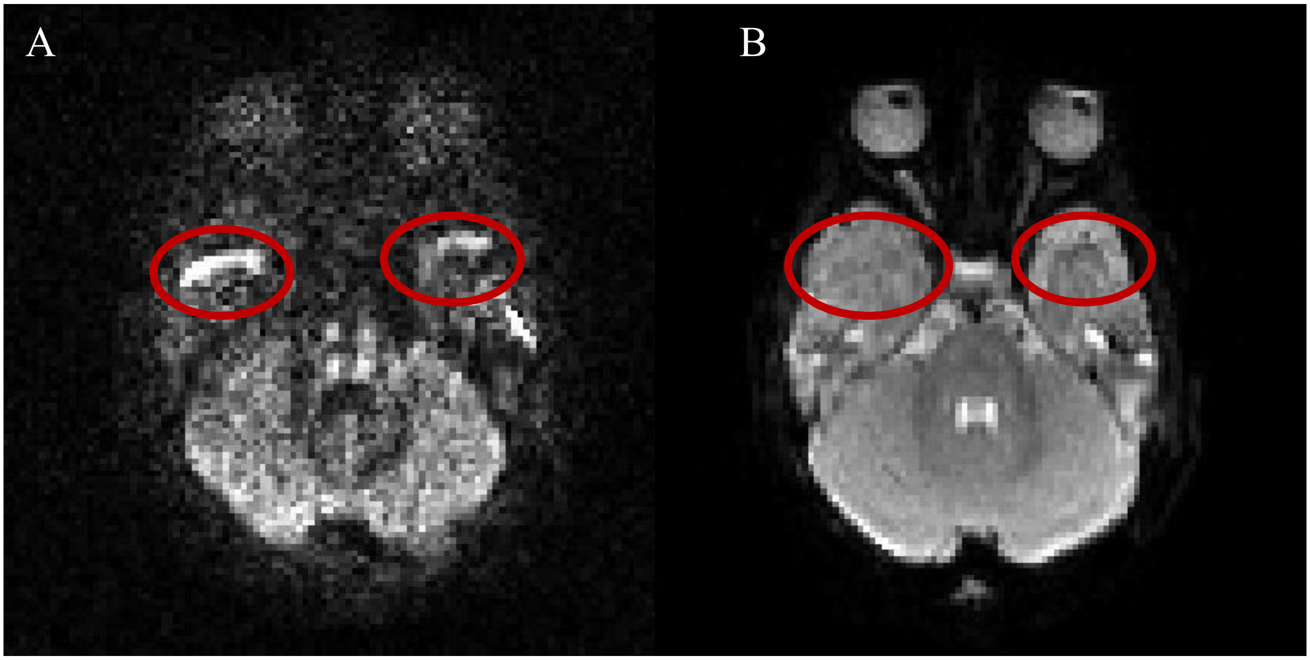

eFigure 1. An example of the susceptibility artifact is seen bilaterally in the temporal poles on high b-value (A) and low b-value (B) DWI.

Tracts	
Arcuate Fasciculus FrontoParietal Left	Corticothalamic Motor Left
Arcuate Fasciculus FrontoParietal Right	Corticothalamic Motor Right
Arcuate Fasciculus FrontoTemporal Left	Corticothalamic Parietal Left
Arcuate Fasciculus FrontoTemporal Right	Corticothalamic Parietal Right
Arcuate Fasciculus TemporoParietal Left	Corticothalamic PreFrontal Left
Arcuate Fasciculus TemporoParietal Right	Corticothalamic PreFrontal Right
Cingulate Gyrus part of the Cingulum Left	Corticothalamic PreMotor Left
Cingulate Gyrus part of the Cingulum Right	Corticothalamic PreMotor Right
Corpus Callosum Body	Corticothalamic Superior Left
Corpus Callosum Genu	Corticothalamic Superior Right
Corpus Callosum Motor	Fornix Left
Corpus Callosum Parietal	Fornix Right
Corpus Callosum PreMotor	Hippocampal part of the Cingulum Left
Corpus Callosum Splenium	Hippocampal part of the Cingulum Right
Corpus Callosum Tapetum	Inferior Fronto-Occipital Fasciculus Left
Corticofugal Motor Left	Inferior Fronto-Occipital Fasciculus Right
Corticofugal Motor Right	Inferior Longitudinal Fasciculus Left
Corticofugal Parietal Left	Inferior Longitudinal Fasciculus Right
Corticofugal Parietal Right	Optic Radiation Left
Corticofugal PreFrontal Left	Optic Radiation Right
Corticofugal PreFrontal Right	Optic Tract Left
Corticofugal PreMotor Left	Optic Tract Right
Corticofugal PreMotor Right	Superior Longitudinal Fasciculus II Left
CorticoSpinal Left	Superior Longitudinal Fasciculus II Right
CorticoSpinal Right	Uncinate Fasciculus Left
	Uncinate Fasciculus Right

eTable 1. Index of all tracts examined in secondary analyses.

eTable 2. Results of Multivariate analyses of variance comparing DTI and NODDI parameters in the tracts of interest between DS and control infants. (****p*-value is <0.0001, ***p*-value is 0.01 - 0.001, **p*-value is 0.05 - 0.01).

Tract	*Test*	*Multivariate Analysis of Variance*				*Cohort*	
		Value	Approx. F	*p*-value	Corrected *p*-value	Exact F	*p*-value
**Corpus Callosum Parietal**	Wilks' Lambda	0.3517668	4.5001	0.00001***	0.00015***	8.5062	0.00001***
	Pillai's Trace	0.7936431	3.7623	0.00001***	0.00015***		
**Corpus Callosum Splenium**	Wilks' Lambda	0.5177167	2.7412	0.0001***	0.0015**	1.9091	0.1028
	Pillai's Trace	0.5654121	2.5681	0.0003***	0.0045**		
**Corpus Callosum Tapetum**	Wilks' Lambda	0.5625489	2.3333	0.0014**	0.021*	5.7214	0.0002***
	Pillai's Trace	0.4940561	2.1702	0.003**	0.045*		
**Corticofugal PreFrontal Left**	Wilks' Lambda	0.5676535	2.386	0.001**	0.015*	1.0374	0.4019
	Pillai's Trace	0.4985278	2.278	0.0016**	0.024*		
**Corticofugal PreFrontal Right**	Wilks' Lambda	0.574091	2.3344	0.0013**	0.0195*	1.369	0.2453
	Pillai's Trace	0.4946746	2.2579	0.0018**	0.027*		
**Corticospinal Left**	Wilks' Lambda	0.5797876	2.2894	0.0017**	0.0255*	6.0233	0.00001***
	Pillai's Trace	0.476954	2.1661	0.003**	0.045*		
**Corticospinal Right**	Wilks' Lambda	0.5869572	2.2046	0.0027**	0.0405*	5.344	0.0003***
	Pillai's Trace	0.4718462	2.1131	0.004**	0.06		
**Inferior Fronto-Occipital Fasciculus Left**	Wilks' Lambda	0.318121	5.081	0.00001***	0.00015***	10.571<br>6	0.00001***
	Pillai's Trace	0.921279	4.6083	0.00001***	0.00015***		
**Inferior Fronto-Occipital Fasciculus Right**	Wilks' Lambda	0.4373586	3.3478	0.00001***	0.00015***	6.7666	0.00001***
	Pillai's Trace	0.6948693	3.1115	0.00001***	0.00015***		
**Inferior Longitudinal Fasciculus Left**	Wilks' Lambda	0.4899746	2.5186	0.0006***	0.009**	5.123	0.0005***
	Pillai's Trace	0.5939683	2.3019	0.0016**	0.024*		
**Inferior Longitudinal Fasciculus Right**	Wilks' Lambda	0.4098067	3.3412	0.00001***	0.00015***	5.5868	0.0002***
	Pillai's Trace	0.75312	3.1545	0.00001***	0.00015***		
**Superior Longitudinal Fasciculus II Left**	Wilks' Lambda	0.2363805	5.4462	0.00001***	0.00015***	12.158<br>6	0.00001***
	Pillai's Trace	1.0818574	4.6713	0.00001***	0.00015***		
**Superior Longitudinal Fasciculus II Right**	Wilks' Lambda	0.3109093	4.0814	0.00001***	0.00015***	10.729<br>6	0.00001***
	Pillai's Trace	0.8199872	3.1459	0.00001***	0.00015***		
**Uncinate Fasciculus Left**	Wilks' Lambda	0.5185115	2.4073	0.001**	0.015*	4.3313	0.0018**
	Pillai's Trace	0.5730366	2.3076	0.0015**	0.0225*		
**Uncinate Fasciculus Right**	Wilks' Lambda	0.3974788	3.6319	0.00001***	0.00015***	6.5693	0.00001***

	Pillai's Trace	0.780231	3.441	0.00001***	0.00015***	

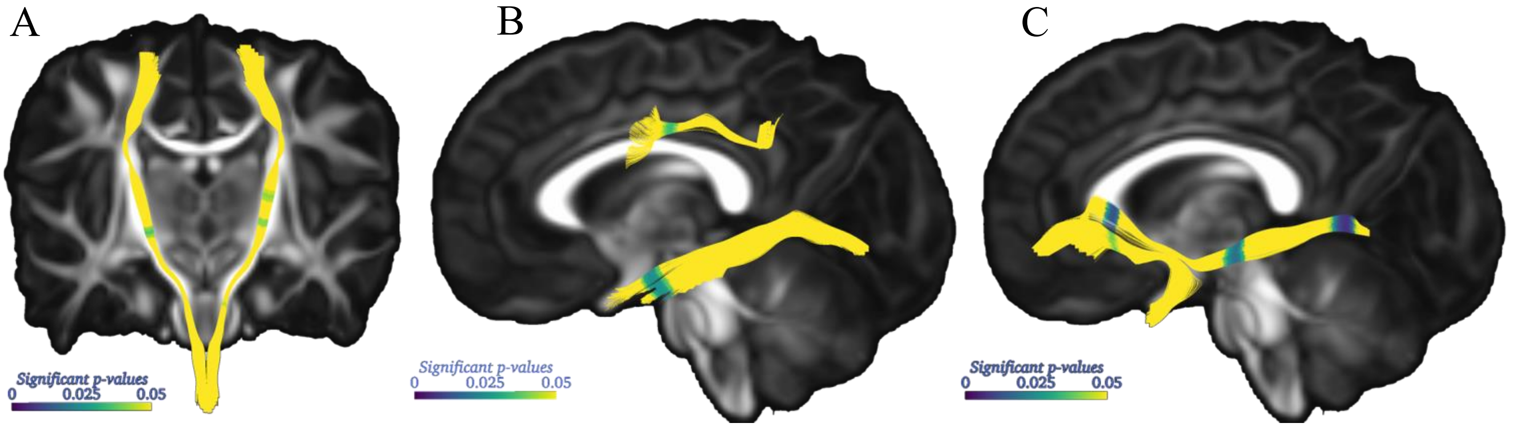

eFigure 2. Visualization of the significant *p*-values along the tracts generated using Slicer. (A) displays significant regions of RD in the bilateral CST, (B) highlights significant RD in the left SLF II and ILF, and (C) shows significant FA in the right IFOF and UNC. Abbreviations: IFOF, inferior fronto-occipital fasciculus; ILF, inferior longitudinal fasciculus; SLF, superior longitudinal fasciculus; CST, corticospinal tract; UNC, uncinate fasciculus. FA, fractional anisotropy; RD, radial diffusivity.
